# Characterization of *Clinostomum* (Digenea: Clinostomidae) spp. in India

**DOI:** 10.1007/s00436-022-07644-y

**Published:** 2022-09-07

**Authors:** Kirti Choudhary, Shailendra Ray, Shokoofeh Shamsi, Nirupama Agrawal

**Affiliations:** 1 Department of Zoology, University of Lucknow, Lucknow, 226007 U.P. India; 2 School of Agricultural, Environmental and Veterinary Sciences, Charles Sturt University, Wagga Wagga, Australia

**Keywords:** Molecular taxonomy, River systems, Wildlife parasitology, Conservation

## Abstract

Platyhelminths belonging to the family Clinostomidae (Digenea) have a worldwide distribution and are known to infect piscivorous birds through their intermediate hosts, usually fish species. In the present study, clinostome metacercariae were collected from fish hosts, including *Channa punctata* (Bloch 1793) (*n* = 25) and *Trichogaster fasciata* Bloch and Schneider 1801 (*n* = 25), from a freshwater system in India. The experimental infection of cattle egrets, *Ardea* (*Bubulcus*) *ibis* Linnaeus 1758, with some of the live metacercariae found in the present study was successful. Live adult parasites were obtained from the buccal cavity of the birds. Both metacercaria and adult specimens were subjected to molecular studies to obtain the sequences of 28S, ITS1, and ITS2 (nuclear rDNA) regions. The parasites were found to belong to three species, *Clinostomum giganticum* Agarwal 1959; *C. piscidium* Southwell and Prashad 1918; and *Euclinostomum heterostomum* (Rudolphi 1809). Phylogenetic analyses of the sequences obtained from the adults and metacercariae established a link between the metacercariae in the fish hosts and adults in the avian host, which is essential to elucidate their partial life cycle and specify morphological characteristics in the metacercarial stage.

**Supplementary information:**

The online version contains supplementary material available at 10.1007/s00436-022-07644-y.

## Introduction

Parasites belonging to the family Clinostomidae (Digenea) have an indirect life cycle. Adult parasites inhabit the oral cavity, pharynx, or esophagus of fish-eating birds, reptiles, and occasionally some mammals (Kamo et al. 1962; Kagei et al. 1988; Kanev et al. 2002). The cercarial stage infects various snail species, and the metacercarial stage is found in several freshwater fish species (Mitchell 1995; Aghlmandi et al. 2018; Caffara et al. 2020; Shamsi et al. 2021b), causing “yellow grub” in them. In the Indian subcontinent, descriptions of metacercariae belong to those of *Clinostomum piscidium* Southwell and Prashad 1918 from *Nandus nandus* (Hamilton 1822) and *Trichogaster fasciata* Bloch and Schneider, 1801 (e.g., Bhalerao 1942; Singh 1959; Pandey and Baugh 1969). Another metacercaria, *C. giganticum* Agarwal, 1959 was found in *Channa punctatus* (Bloch 1793) at Jabalpur (Madhya Pradesh) (Agarwal 1960).

Unlike morphological data on clinostomid parasites in India, our knowledge of their genetic characterization is poor. Nowadays, the use of sequence data to validate the taxonomic status of parasites or specifically identify immature and larval stages of parasites and elucidate their life cycle is common and has proved useful (Nolan and Cribb 2005; Shamsi et al. 2011). Ribosomal DNA (rDNA) has frequently been exploited as a prospective marker for phylogenetic studies. The present study aimed to genetically characterize metacercariae belonging to the family Clinostomidae from freshwater fish in India in order to specifically identify them.

## Materials and methods

The protocols for this study were based on the animal ethics guidelines of the University of Lucknow, India, Protocol number: LU/ZOOL/SR-SM/Res/NA/11–2014. A total of 50 fish belonging to two species, *Channa punctatus* (*n* = 25) and *Trichogaster fasciata* (*n* = 25) were purchased from fishermen. They were caught in Naya Tal at Barabanki (26° 55ʹ N, 81° 11ʹ E), U.P., India. They were transported to the Helminthes Laboratory of Lucknow University in aerated polyethylene and maintained in aquaria under proper aeration. They were fed on commercial pellets containing macronutrients (soybean meal, wheat, rice flour) and vitamins (A, C, D3, E). The fish were euthanized by overdosing them with anesthetics (trichloro-tertiary-butyl-alcohol). They were examined for the presence of parasites according to standard protocols (Fernando et al. 1972).

Morphological and molecular analyses were performed to determine the species of the parasites. Metacercariae were recovered from fish and if encysted, a sharp needle was used to remove them. After fixing them in ethanol, a small section was cut from the parasite for DNA extraction. The parasites were then fixed between two glass slides with 70% ethanol (Aghlmandi et al. 2018; Shamsi et al. 2021b). The infected organs, number of cysts per fish and other important data such as the fish’s species and locality were recorded. The prevalence (P), mean intensity (MI), and mean abundance (MA) of parasites were calculated according to Bush et al. (1997).

To obtain adult worms, two cattle egrets (*Bubulcus ibis* Linnaeus, 1758) were first treated with Praziquantel. The buccal cavity and fecal matter of each egret were examined over 4 days to ensure there was no existing infection. Then, one egret was fed with six live metacercaria of *C. pisicidium* with the help of glass droppers. The buccal cavities were regularly examined for the adult worms as well as the faeces for eggs. After 8 days, five worms were found, firmly attached by their suckers to the mucous membrane of the buccal cavity of the bird. Adult parasites were then carefully collected from the buccal cavity with the aid of forceps without any harm to the bird. Then, the same bird was infected with six live metacercaria of *C. giganticum* and similarly only five adult worms were collected after 8 days. The second bird was fed with six live *Euclinostomum* metacercariae and five adult worms were collected. All worms were subjected to both morphological and molecular analyses, as mentioned above. Note that prior to feeding to the birds, the metacercaria were separated into different morphotypes, based on overall morphological characteristics as described in the “Discussion” section, and then identification was confirmed following sequences were obtained.

Both the metacercariae and adults were fixed in 70% ethanol, stained in aqueous aceto-alum carmine, dehydrated in graded ethanol series, cleared in clove oil, and mounted in DPX. Figures were made using a drawing tube attached to a phase contrast microscope (Olympus CX-41, Tokyo, Japan). Measurements were taken in mm with the aid of an ocular micrometer. Voucher specimens were deposited in the Helminthological Collection of the Zoological Survey of India, Kolkata, under accession numbers W10418/1 to W10422/1.

Genomic DNA was individually extracted by Qiagen’s DNeasy Tissue Kit (Qiagen Hilden, Germany), following the manufacturer’s instructions. PCR was performed using primer sets and conditions detailed in previous studies (Bowles et al. 1995; Mollaret et al. 2000) to amplify the ITS-1, ITS-2, and 28S regions. The purified PCR products were subjected to sequencing. Sequencing was carried out by Genomics Crop-Xceleris, Bangalore, using an automated sequencer (model name 3130 × 1/3130x/GA-1203–019). The sequences obtained were deposited in GenBank under accession numbers KY247140-1, KY273277-8, KY29051-2, KY304778-80, KY311833-4, KY312846-8, and KY319339-41. An alignment was constructed by Clustal W (Thompson et al. 1994). Phylogenetic trees were generated using MEGA 6 (Tamura et al. 2013) and analyzed using the neighbor-joining (NJ) and maximum likelihood (ML) methods for each dataset (28S, ITS1, and ITS2). Potential species were distinguished by clustering in the NJ method of the best nucleotide substitution model estimated by the “Kimura 2 parameter model.” More complicated models may sometimes yield inconsistent results when large numbers of sequences are compared. The best model (general time reversible model) for ML analysis was selected with a gamma distribution of rates and proportion of invariant sites (GTR + G + I), which provide the best fit to the data. All positions containing gaps and missing data were eliminated. The reliability of internal branches in all the trees was estimated using the bootstrap method with 1000 replicates (Hillis et al. 1993).

## Results

The prevalence, mean intensity, and mean abundance of metacercariae found in the present study are shown in Table 1. Both fish species were found to be infected with metacercaria of *Clinostomum*. Of six metacercariae fed to the birds, five adults were recovered after 8 days (success rate of 83%). For all three parasite species, six metacercaria were fed to the birds but only 5 adults were recovered after eight days (success rate of 83%). The presence of eggs confirmed the maturity of the worms collected from the birds. It was observed that after feeding the birds with metacercariae, they were not visible in the buccal cavity, assuming they entered the gut of the birds. The birds’ buccal cavities were regularly checked by opening their beaks. Worms first appeared after 7 days but were collected after 8 days.

**Table 1:** Prevalence, mean intensity, and relative densities of metacercariae found in the present study

Fish host	Parasite	No of host examined	No of infected Fish	Prevalence (%)	No. of Parasite	Mean intensity	Mean abundance
*Channa punctatus*	*C. giganticum*	25	5	20	10	2	0.40
*Channa punctatus*	*E. heterostomum*	25	16	64	96	6	3.84
*Trichogaster fasciata*	*C. piscidium*	25	18	72	72	4	2.88

The parasites were identified to species level based on the morphology of the adult worms collected from the birds, and then the sequences of both adults and metacercaria were obtained. Metacercariae were identified based on the matching sequence with adult specimens.

**Fig. 1:**
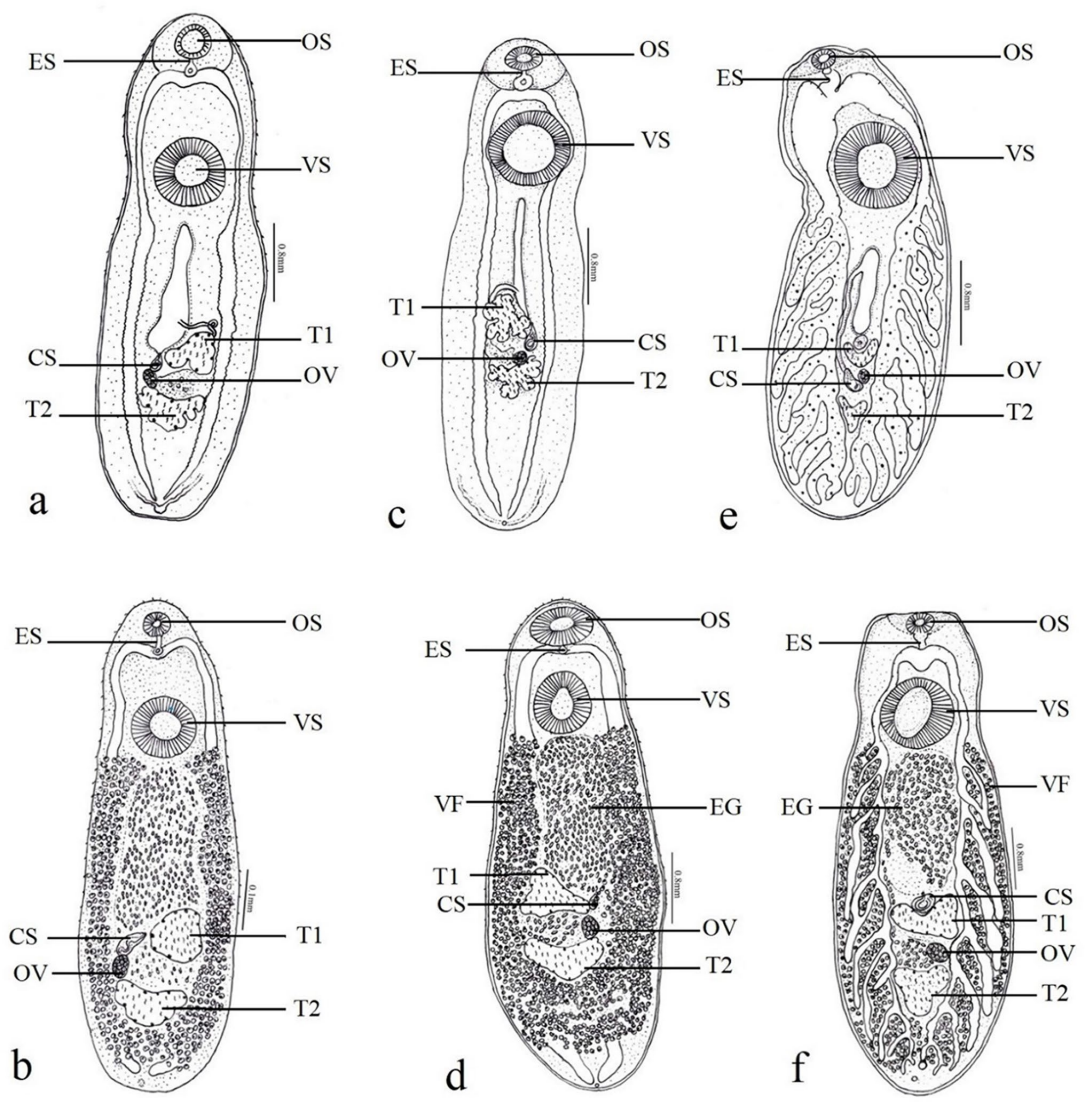
Metacercaria and adult *C. giganticum* (**a** and **b**, respectively), metacercaria and adult *C. piscidium* (**c** and **d**, respectively), and metacercaria and adult *E. heterostomum* (**e** and **f**, respectively). OS oral sucker, ES esophagus, VS ventral sucker, T1 anterior testis, T2 posterior testis, OV ovary, CS cirrus sac, VF vitelline follicles, EG eggs

A total of three species were found in the present study, *C. giganticum*, *C. piscidium*, and *Euclinostomum heterostomum* (Fig. 1)*.* Metacercariae of *C. giganticum* and *E. heterostomum* were found in *Channa punctatus* whereas *C. piscidium* metacercaria was found in *Trichogaster fasciata.* No mixed infection was observed in the individual of *Channa punctatus* examined in the present study. Euclinostome metacercaria were found beneath the operculum within the branchial chamber of the host, whereas clinostome metacercariae were found in the excysted form in the body cavity of the host attached to visceral organs.

Metacercaria and adult *C. giganticum* both had a spinose body, a subterminal oral sucker smaller than the ventral sucker, a short tubular esophagus, and intestinal caecae up to the hind end of body, forming shoulders. There were two testes with a crenated margin in the metacercaria, but smooth in adult *C. giganticum*. The cirrus sac in both metacercaria and adults was elongated to oval, opening into the genital atrium. Other characteristics for both developmental stages included a tubular vesicula seminalis, a small ovary between the two testes, vitellaria consisting of small vitelline follicles distributed from the level of the posterior border of the ventral sucker and extending to the posterior extremity of the body (adult) and a V-shaped excretory bladder.

Metacercaria and adult *C. piscidium* had a linguiform body with spines only in the anterior half, a small subterminal oral sucker, smaller than the ventral sucker in metacercaria but almost equal in adults, a pseudo-pharynx, smooth intestinal caecae, forming shoulders in the adult, gonads in the middle third of the body, two testes, deeply lobed with a crenated margin in the metacercaria but with a smooth border in the adults, cirrus sac, ovoidal ovary between the two testes, large uterus in the middle line, extending anterior to the lower margin of the ventral sucker, opening posteriorly into the genital atrium, a genital pore at the middle level of the anterior testis, an excretory U-shaped bladder and excretory pore at the posterior end.

Metacercaria and adult *E. heterostomum* had aspinous adult but thin, spinous, fibrous, round to oval shape metacercaria with a subterminal oral sucker smaller than the ventral sucker. There was a short esophagus in both metacercaria and adults with ten to eleven lateral diverticles in the latter form, lobed testes (anterior curved and U-shaped; posterior Y and triangular shaped in metacercaria and adult forms, respectively), an elongated-tubular cirrus sac between two limbs of the anterior testis, a saccular, vesicula seminalis, a globular ovary between the two testes, and a subterminal excretory pore.

Measurements of taxonomically important features are provided in Table 2.

**Table 2:** Comparative measurements of metacercaria (M) and adult (A) of specimens in the present study (*n* = 10) with previous studies

	*C. piscidium*				*C. gignaticum*			
Reference	Pandey (1973)	Present study	Pandey (1973)	Present study	Agrawal, 1959	Present study	Agrawal, 1959	Present study
Developmental stage	M	M	A	A	M	M	A	A
Body size	2.46–3.92 × 0.85–1.22	4.32–4.85 × 1.28–1.32	4.124–4.87 × 1.11–1.93	6.10–6.52 × 2.30–2.52	3.87–7.72 × 1.35–2.42	7.20–7.90 × 2.31–2.62	8.35–14.1 × 2.85–4.5	8.32–9.20 × 1.08–2.3
Oral sucker	0.05–0.12	0.20–0.22 × 0.35–0.38	0.18–0.22	0.42–0.45 × 0.71–0.74	0.28–0.59 × 0.33–0.60	0.52–0.59 × 0.54–0.56	0.35–0.6 × 0.45–0.9	0.30–0.41 × 0.42–0.52
Ventral sucker	0.51–0.62	0.70–0.72 × 0.73–0.76	0.48–0.51	0.72–0.76 × 0.68–0.71	0.73–0.99 × 0.73–1.12	0.95–1.20 × 0.98–1.23	1.0–1.5 × 1.10–1	1.03–1.05 × 0.82–1.0
Esophagus	0.05–0.06	–	0.10–0.12	0.01–0.02	0.06–0.08	0.06–0.08	–	0.07–0.09
Anterior testis	0.10–0.12 × 0.18–0.23	0.42–0.46 × 0.41–0.43	0.25–0.36 × 0.62–0.67	0.52–0.56 × 0.78–0.82	0.52–0.70 × 0.45–0.51	0.62–0.65 × 0.73–0.76	0.8–1.5 × 0.6–1.35	0.72–1.11 × 0.80–0.94
Posterior testis	0.11–0.28 × 0.32–0.35	0.48–0.51 × 0.23–0.26	0.21–0.37 × 0.58–0. 61	0.36–0.38 × 0.91–0.96	0.42–0.78 × 0.69–0.82	0.96–1.20 × 0.43–0.46	0.65–1.05 × 1.0	0.52–0.64 × 1.20–1.10
Cirrus sac	0.30–0.32 × 0.21–0.34	0.23–0.25 × 0.10–0.12	0.18–0.27 × 0.28–0.27	–	0.22–0.64 × 0.13–0.22	0.18–0.23 × 0.12–0.14	0.565–0.721 × 0.273–0.39	0.23–0.53 × 0.11–0.38
Ovary	0.12–0.16	0.10–0.12 × 0.06–0.08	0.18–0.27	0.21–0.24 × 0.18–0.23	0.16–0.22 × 0.12–0.19	0.23–0.25 × 0.14–0.16	0.45–0.75 × 0.3–0.45	0.22–0.30 × 0.08–0.11
Eggs	–	–	0.08–0.09 × 0.04–0.05	0.04–0.06 × 0.03–0.04	–	–	0.112–0.124 × 0.072–0.078	0.04–0.09 × 0.04–0.06

*E. heterostomum*				
Reference	Jhansilakshmibai & Madhavi, 1997	Present study	Jhansilakshmibai & Madhavi, 1997	Present study
Developmental stage	M	M	A	A
Body size	1.83–4.53 × 0.89–1.62	4.20–4.80 × 1.20–1.70	4.80–9.82 × 1.84–3.22	7.30–7.72 × 2.33–2.55
Oral sucker	0.42–1.68 × 0.50–2.17	0.18–0.20 × 0.18–0.20	1.92–3.04 × 2.24–3.52	0.36–0.39 × 0.25–0.28
Ventral sucker	0.70–0.92 × 0.70–0.91	0.76–0.80 × 0.84–0.87	9.60–10.31 × 9.60–10.34	0.80–1.0 × 0.78–0.10
Esophagus	–	–	–	0.07–0.08 × 0.08–0.12
Anterior testis	1.26–3.14 × 1.50–4.91	0.28–0.30 × 0.04–0.06	5.40–9.60 × 5.60–10.37	0.92–0.95 × 0.78–0.80
Posterior testis	1.11–3.70 × 1.45–4.20	0.22–0.25 × 0.21–0.23	4.16–8.64 × 4.32–8.96	0.74–0.78 × 0.58–0.60
Cirrus sac	1.25–1.71 × 1.20–1.85	0.78–0.82 × 0.12–0.15	–	–
Ovary	0.52–1.36 × 0.50–1.46	0.18–0.21 × 0.08–0.10	3.20–4.32 × 3.04–4.08	0.21–0.23 × 0.28–0.32
Eggs	–	﻿–	–	0.07–0.11 × 0.05–0.06

*Clinostomum giganticum* and *C. pisicidium* can be morphologically differentiated based on the distance between the ventral sucker and oral sucker and the width of the ventral sucker, as well as deep, multilobed testes with a crenated margin in the metacercaria of the latter species, whereas almost triangular testes with less digitation in the metacercaria of the former species. However, as adults, in both species, the testes have a smooth border. The position of the genital pore also varies in both species. In C*. giganticum*, the genital pore opens at the left external margin of the anterior testis, both in metacercaria and adult forms, whereas in adult *C. pisicidium*, the genital pore opens at the right side, slightly away from the external margin of the anterior testis, and in metacercariae, it opens close to the external margin to anterior testis in the middle of the body. The shape of the excretory bladder also varies; it is V- or Y-shaped in *C. giganticum*; however, it is U-shaped in *C. piscidium*. The extension of intestinal caeca may be better visualized in both the metacercariae, which can be concealed by vitellaria in adults.

The 28S, ITS1, and ITS2 sequences of both metacercariae and adults of *Clinostomum* species (*C. giganticum* and *C. piscidium*) and *E. heterostomum* were consistent and identical with their respective adults. The phylogenetic relationship between parasite taxa in the present study and closely related taxa is shown in the Supplementary Figure. Panels b and c in the Supplementary Figure, which are trees built based on the ITS regions, show that taxa included in the tree are resolved from one another, whereas in panel a, a tree built based on 28S sequences, adults, and metacercaria of *C. giganticum* are grouped together along with metacercaria and adults of *C piscidium*, which suggests ITS-1 and ITS-2 regions may be more reliable for interspecific differentiation of *Clinostomum* spp.

## Discussion

Parasites belonging to the family Clinostomidae are cosmopolitan, potentially zoonotic flukes that have been poorly studied in India using molecular taxonomic tools. In particular, morphological differences between the different developmental stages of the parasites have made their specific identification more challenging. Therefore, it has been recommended that morphological identification be combined with molecular support to ensure increased accuracy in identifying *Clinostomum* species (Briosio-Aguilar et al. 2019), which is what has been done in the present study. We provided detailed morphological descriptions for the identification of the metacercaria and adult stage of *C. giganticum*, *C. piscidium*, and *E. heterostomum* along with associated sequence data, which can be used in future studies on diagnosis, conservation management plans for freshwater systems, and for public health purposes in India. Although *C. complanatum* has been widely known as a zoonotic parasite (Park et al. 2009), it should be noted that many medical reports are based on this assumption rather than providing evidence on the identity of the parasite (Rahmati et al. 2020). Therefore, the occurrence of metacercariae in their infectious stage in edible fish in the present study should be considered a potential risk.

Clinostomid parasites have low host specificity and can infect a diversity of hosts, damaging and impairing them and, in severe cases, causing death (Sutili et al. 2014; Aghlmandi et al. 2018; Montes et al. 2020; Shamsi et al. 2021b). Although in the present study we examined only two species, clearly more work is needed to investigate the extent of the infection in other fish species and the health impact on their hosts.

The natural definitive hosts are fish-eating birds (Shamsi et al. 2013, 2021a; Rosser et al. 2018), but the link between metacercaria and adult stages is usually difficult to establish using solely morphological criteria (Jousson et al. 1998). The present study linked specifically indistinguishable metacercariae of *Clinostomum* spp. and *Euclinostomum* sp. with their adults, which can be useful for future studies. During the transformation of metacercariae into an adult, a few morphological differences arise, including the shape and size of the body, and ratio of the suckers, and especially, the organization of the genital complex. The keynote feature of the family Clinostomidae is the unarmed cuticle (Kanev et al. 2002), whereas the cuticular spines were observed in the present study. It is noteworthy that during the staining procedure, most of the cuticular spines can be lost and thus no longer detected in a permanent slide (Caffara et al. 2014a, 2014b).

Our results suggest that the three species examined in the present study might be morphologically distinguishable. Adult *C. giganticum*, compared to *C. piscidium*, have a longer esophagus, show less digitation in the testes and have a larger body size. The ratio of the oral sucker to ventral sucker size is one third to one half in the former species, whereas it is one half to the same size in the latter species. The most distinguishing characteristic of *E. heterostomum* was its branched caecae. Metacercaria and adult *C. piscidium* show a close resemblance in terms of the position of oral and ventral suckers, the extension of intestinal caeca, and the position of the genital complex, except for the smooth margin of the testes. Similarly, metacercaria of *E. heterostomum* and its adult are almost alike, except for a few minor differences. For all taxa found in the present study, the comparative morphology of specimens was nearly identical to the original description, with minor differences, which could be due to different fixation and mounting techniques employed.

Our results suggest that ITS-1 and ITS-2 sequences might be more useful for distinguishing between closely related taxa belonging to the family Clinostomidae than 28S sequences. This has also been suggested in previous studies (Curran et al. 2006; Lotfy et al. 2010; Phalee and Wongsawad 2014). The monophyletic origin of *Euclinostomum* has also been indicated in previous findings (Senapin et al. 2014; Caffara et al. 2016), as illustrated in ITS2 in the present study but not with other markers. In this study, we report for the first time the link between metacercariae and adults of two species of *Clinostomum* and *Euclinostomum*.

## Supplementary information

Below is the link to the electronic supplementary material.

Supplementary file1 (DOCX 198 KB)

## References

[CR1] Agarwal S (1960). Studies on the morphology, systematics and life history of *Clinostomum giganticum* n. sp. (Trematoda: Clinostomatidae). Indian J Helminthol.

[CR2] Aghlmandi F, Habibi F, Afraii MA, Abdoli A, Shamsi S (2018). Infection with metacercaria of *Clinostomum complanatum* (Trematoda: Clinostomidae) in freshwater fishes from Southern Caspian Sea Basin. Rev Méd Vét.

[CR3] Bhalerao G (1942) Some metacercarial forms of Clinostomatidae (Trematoda) from India. Proc Indian Acad Sci-Section B Vol 16. Springer India, p 67–71

[CR4] Bowles J, Blair D, McManus DP (1995). A molecular phylogeny of the human schistosomes. Mol Phylogenet Evol.

[CR5] Briosio-Aguilar R, García-Varela M, Hernández-Mena D, Rubio-Godoy M, De León GP-P (2019). Morphological and molecular characterization of an enigmatic clinostomid trematode (Digenea: Clinostomidae) parasitic as metacercariae in the body cavity of freshwater fishes (Cichlidae) across Middle America. J Helminthol.

[CR6] Bush AO, Lafferty KD, Lotz JM, Shostak AW (1997). Parasitology meets ecology on its own terms: Margolis et al revisited. J Parasitol.

[CR7] Caffara M, Bruni G, Paoletti C, Gustinelli A, Fioravanti M (2014). Metacercariae of *Clinostomum complanatum* (Trematoda: Digenea) in European newts *Triturus carnifex* and *Lissotriton vulgaris* (Caudata: Salamandridae). J Helminthol.

[CR8] Caffara M, Davidovich N, Falk R, Smirnov M, Ofek T, Cummings D, Gustinelli A, Fioravanti ML (2014). Redescription of *Clinostomum phalacrocoracis* metacercariae (Digenea: Clinostomidae) in cichlids from Lake Kinneret Israel. Parasite.

[CR9] Caffara M, Locke SA, Cristanini C, Davidovich N, Markovich MP, Fioravanti ML (2016). A combined morphometric and molecular approach to identifying metacercariae of *Euclinostomum heterostomum* (Digenea: Clinostomidae). J Parasitol.

[CR10] Caffara M, Locke SA, Echi PC, Halajian A, Luus-Powell WJ, Benini D, Tedesco P, Fioravanti ML (2020). A new species of *Clinostomum* Leidy, 1856 based on molecular and morphological analysis of metacercariae from African siluriform fishes. Parasitol Res.

[CR11] Curran SS, Tkach VV, Overstreet RM (2006). A review of Polylekithum Arnold, 1934 and its familial affinities using morphological and molecular data, with description of *Polylekithum catahoulensis* sp. nov. Acta Parasitol.

[CR12] Fernando CH, Furtado JI, Gussev AV, Hanek G, Kakonge SA (1972) Methods for the study of freshwater fish parasites. University of Waterloo series. No 12

[CR13] Hillis DM, Bull JJ, Felsenstein J, Kishino H (1993). An empirical test of bootstrapping as a method for assessing confidence in phylogenetic analysis. Is there something wrong with the bootstrap on phylogenies? A reply to Hillis and Bull. Syst Biol.

[CR14] Jousson O, Bartoli P, Zaninetti L, Pawlowski J (1998). Use of the ITS rDNA for elucidation of some life-cycles of Mesometridae (Trematoda, Digenea). Int J Parasitol.

[CR15] Kagei N, Yanohara Y, Uchikawa R, Sato A (1988) Natural infection with *Clinostomum complanatum* Rud. 1819 in the birds of southern Japan. Jpn J Parasitol 37(4):254–257

[CR16] Kanev I, Radev V, Fried B (2002) Family Clinostomidae Lühe, 1901. In: Keys to the Trematoda, vol 1, Editors: Gibson DI, Jones A, Bray RA. CAB International and the Natural History Museum, Wallingford, UK:113–120

[CR17] Kamo H, Ogino K, Hatsushika R (1962). A unique infection of man with *Clinostomum* sp., a small trematode causing acute laryngitis. Yonago Acta Med.

[CR18] Lotfy WM, Brant SV, Ashmawy KI, Devkota R, Mkoji GM, Loker ES (2010). A molecular approach for identification of paramphistomes from Africa and Asia. Vet Parasitol.

[CR19] Mitchell A (1995). Yellow grubs and other problems associated with aquatic birds. Aquac Mag.

[CR20] Mollaret I, Lim LHS, Justine J-L (2000). Phylogenetic position of the monogeneans *Sundanonchus*, *Thaparocleidus*, and *Cichlidogyrus* inferred from 28S rDNA sequences. Int J Parasitol.

[CR21] Montes M, Plaul S, Croci Y, Waldbillig M, Ferrari W, Topa E, Martorelli S (2020) Pathology associated with three new *Clinostomum* metacercariae from Argentina with morphological and DNA barcode identification. J Helminthol 94(e148):1–11. 10.1017/S0022149X20000292 10.1017/S0022149X20000292 32364092

[CR22] Nolan MJ, Cribb TH (2005). The use and implications of ribosomal DNA sequencing for the discrimination of digenean species. Adv Parasitol.

[CR23] Pandey K, Baugh S (1969) Studies on clinostome metacercariae. II. A restudy of *Clinostomum piscidium* from metacercaria and adult. Zool Anz 183(5/6):463–480

[CR24] Park CW, Kim JS, Joo HS, Kim J (2009). A human case of *Clinostomum complanatum* infection in Korea. Korean J Parasitol.

[CR25] Phalee A, Wongsawad C (2014). Prevalence of infection and molecular confirmation by using ITS-2 region of *Fasciola gigantica* found in domestic cattle from Chiang Mai province, Thailand. Asian Pac J Trop Med.

[CR26] Rahmati AR, Kiani B, Afshari A, Moghaddas E, Williams M, Shamsi S (2020). World-wide prevalence of *Anisakis* larvae in fish and its relationship to human allergic anisakiasis: a systematic review. Parasitol Res.

[CR27] Rosser TG, Baumgartner WA, Alberson NR, Noto TW, Woodyard ET, King DT, Wise DJ, Griffin MJ (2018) *Clinostomum poteae* n. sp. (Digenea: Clinostomidae), in the trachea of a double-crested cormorant *Phalacrocorax auritus* Lesson, 1831 and molecular data linking the life-cycle stages of *Clinostomum album* Rosser, Alberson, Woodyard, Cunningham, Pote & Griffin, 2017 in Mississippi, USA. Syst Parasitol 95(6):543–566 10.1007/s11230-018-9801-5 29855982

[CR28] Senapin S, Phiwsaiya K, Laosinchai P, Kowasupat C, Ruenwongsa P, Panijpan B (2014). Phylogenetic analysis of parasitic trematodes of the genus *Euclinostomum* found in trichopsis and betta fish. J Parasitol.

[CR29] Shamsi S, Gasser RB, Beveridge I (2011). Mutation scanning-coupled sequencing of nuclear ribosomal DNA spacers (as a taxonomic tool) for the specific identification of different *Contracaecum* (Nematoda: Anisakidae) larval types. Mol Cell Probes.

[CR30] Shamsi S, Halajian A, Tavakol S, Mortazavi P, Boulton J (2013). Pathogenicity of *Clinostomum complanatum* (Digenea: Clinostomidae) in piscivorous birds. Res Vet Sci.

[CR31] Shamsi S, Barton DP, Day S, Masiga J, Zhu X, McLellan M (2021a) Characterization of *Clinostomum* sp. (Trematoda: Clinostomidae) infecting cormorants in south-eastern Australia. Parasitol Res 120:2793–2803. 10.1007/s00436-021-07246-0 10.1007/s00436-021-07246-0 34331138

[CR32] Shamsi S, Day S, Zhu X, McLellan M, Barton DP, Dang M, Nowak BF (2021b) Wild fish as reservoirs of parasites on Australian Murray Cod farms. Aquaculture 539:736584. 10.1016/j.aquaculture.2021.736584

[CR33] Singh R (1959). Studies on the morphology and life history of *Clinostomum piscidium* Southwell and Prashad, 1918 (Trematoda: Clinostomatidae). Proc Nat Acad Sciences India, Section B Biol Sci.

[CR34] Sutili FJ, Gressler LT, de Pelegrini LFV (2014) *Clinostomum complanatum* (Trematoda, Digenea): a parasite of birds and fishes with zoonotic potential in southern Brazil. A Review. Revista Brasileira de Higiene e Sanidade Animal: RBHSA 8(1):99–114

[CR35] Tamura K, Stecher G, Peterson D, Filipski A, Kumar S (2013) MEGA6: Molecular evolutionary genetics analysis version 6.0. Mol Biol Evol 30(12):2725–2729 10.1093/molbev/mst197 10.1093/molbev/mst197 PMC3840312 24132122

[CR36] Thompson JD, Higgins DG, Gibson TJ (1994). CLUSTAL W: improving the sensitivity of progressive multiple sequence alignment through sequence weighting, position-specific gap penalties and weight matrix choice. Nucleic Acids Res.

