# Supplementary material for: Characterization of Clinostomum (Digenea: Clinostomidae) spp. in India
Source: Parasitol Res. 2022 Sep 7;121(11):3083–9. doi: 10.1007/s00436-022-07644-y (PMC9556428; doi:10.1007/s00436-022-07644-y)
Supplement: Supplementary file 1 — Supplementary file1 (DOCX 198 KB) [file 436_2022_7644_MOESM1_ESM.docx]

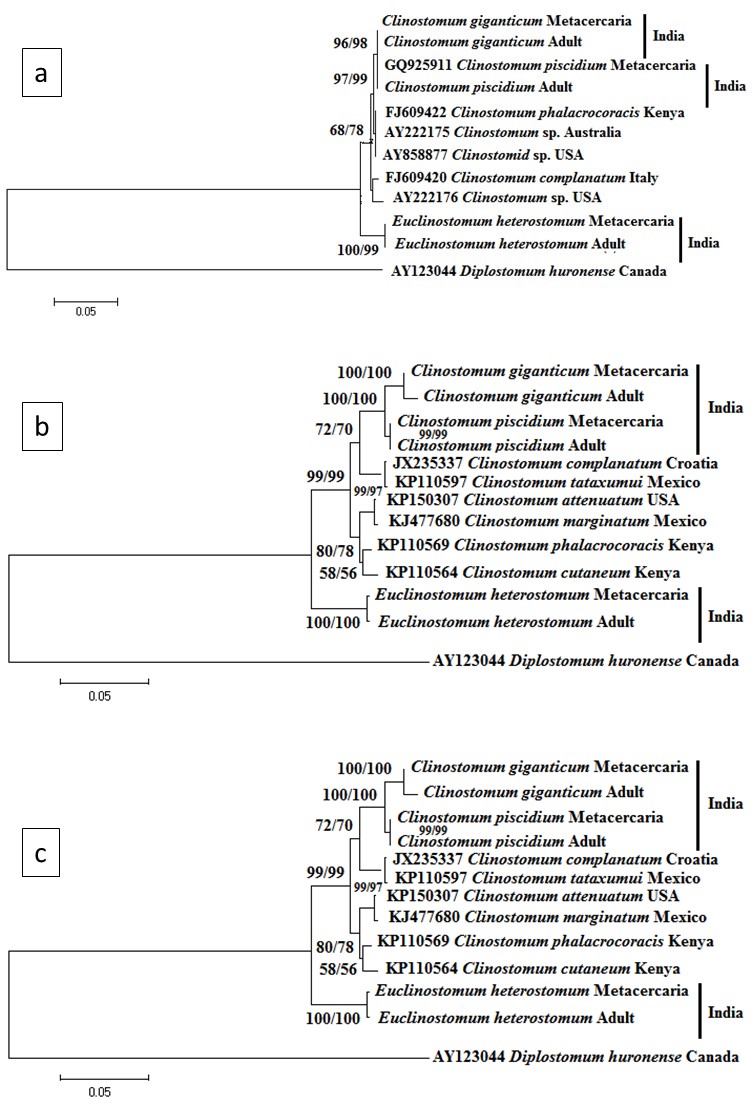


Supplementary Figure. Phylogenetic tree depicting relationships between taxa found in the present study and those found elsewhere inferred from 28S (a), ITS-1 (b) and ITS-2 (c) rRNA gene sequence data. NJ and ML tree from the phylogenetic analysis of 28S (a), ITS-1 (b) and ITS-2 (c) rRNA gene sequence dataset of both metacercariae and adults of *Clinostomum* species (*C. giganticum* and *C. piscidium*) and *E. heterostomum* using members of the family Clinostomidae). Numbers preceding the taxa are GenBank accession numbers for their28S, ITS-1 and ITS-2 rRNA gene sequences. Numbers at the internodes are NJ bootstrap values (above) and ML bootstrap values (below). *Diplistomum huronense* (AY123044) was employed as an outgroup.
